# Retrospective Analyses of Porcine Circovirus Type 3 (PCV-3) in Switzerland

**DOI:** 10.3390/v16091431

**Published:** 2024-09-07

**Authors:** Giuliana Rosato, Grace Makanaka Makoni, Àlex Cobos, Marina Sibila, Joaquim Segalés, Hanna Marti, Barbara Prähauser, Frauke Seehusen

**Affiliations:** 1 Institute of Veterinary Pathology, Vetsuisse Faculty, University of Zurich, 8057 Zurich, Switzerland; g.rosato@access.uzh.ch (G.R.); gracemakanaka.makoni@uzh.ch (G.M.M.); hanna.marti@uzh.ch (H.M.); barbara.praehauser@uzh.ch (B.P.); 2 Unitat Mixta d’Investigació IRTA UAB en Sanitat Animal, Centre de Recerca en Sanitat Animal (CReSA), Campus de la Universitat Autònoma de Barcelona (UAB), 08193 Bellaterra, Spain; alex.cobos@irta.cat (À.C.); marina.sibila@irta.cat (M.S.); joaquim.segales@irta.cat (J.S.); 3 IRTA, Programa de Sanitat Animal, Centre de Recerca en Sanitat Animal (CReSA), Campus de la Universitat Autònoma de Barcelona (UAB), 08193 Bellaterra, Spain; 4 WOAH Collaborating Centre for the Research and Control of Emerging and Re-Emerging Swine Diseases in Europe (IRTA-CReSA), 08193 Bellaterra, Spain; 5 Departament de Sanitat i Anatomia Animals, Facultat de Veterinària, Campus de la Universitat Autònoma de Barcelona (UAB), 08193 Bellaterra, Spain

**Keywords:** porcine circovirus 3 (PCV-3), PCV-3 systemic disease (PCV-3-SD), vascular lesions, real-time quantitative polymerase chain reaction (qPCR), histopathology, tissue microarray (TMA), in situ hybridization (ISH), diagnostic

## Abstract

Porcine circovirus 3 (PCV-3) has emerged as a significant pathogen affecting global swine populations, yet its epidemiology and clinical implications remain incompletely understood. This retrospective study aimed to investigate the prevalence and histopathological features of PCV-3 infection in pigs from Switzerland, focusing on archival cases of suckling and weaner piglets presenting with suggestive lesions. An in-house qPCR assay was developed for detecting PCV-3 in frozen and formalin-fixed paraffin-embedded tissues, enhancing the national diagnostic capabilities. Histopathological reassessment identified PCV-3 systemic disease (PCV-3-SD) compatible lesions in 19 (6%) of archival cases, with 47% testing positive by qPCR across various organs. Notably, vascular lesions predominated, particularly in mesenteric arteries, heart, and kidneys. The study confirms the presence of PCV-3 in Switzerland since at least 2020, marking the first documented cases within the Swiss swine population. Despite challenges in in situ hybridization validation due to prolonged formalin fixation, the findings indicate viral systemic dissemination. These results contribute to the understanding of PCV-3 epidemiology in Swiss pigs, emphasizing the need for continued surveillance and further research on its clinical implications and interaction with host factors. Our study underscores the utility and limitations of molecular techniques in confirming PCV-3 infections.

## 1. Introduction

Circoviruses are small, single-stranded, circular, non-enveloped DNA viruses that infect a broad spectrum of animals. To date, four distinct porcine circoviruses (PCVs) have been identified and designated numerically based on the order of their chronological discovery: PCV-1, PCV-2, PCV-3, and PCV-4.

Porcine circovirus 3 (PCV-3) was first reported in the United States in 2015 by metagenomic analysis in sows displaying porcine dermatitis and nephropathy syndrome-like lesions (PDNS) and reproductive disorders. Additionally, the viral genome was detected in piglets presenting myocarditis and systemic vascular inflammation [1,2]. Since its initial identification, PCV-3 has been identified worldwide, including in Europe [3,4,5], Africa [6,7,8], Asia [9,10,11], and South America [12,13] in healthy animals and in pigs with different clinical presentations, highlighting its global presence. Furthermore, the genetic diversity of PCV-3 strains has been investigated, revealing the existence of distinct phylogenetic clustering [14,15]. The various clinical and pathological presentations associated with PCV-3 infection were proposed to encompass mainly reproductive disorders, PDNS [1], and multisystemic inflammatory disease [2]. Viral DNA has also been detected in pigs suffering from pulmonary disorders [16,17,18], diarrhea [16,19], and neurological signs [20,21]. Among all published studies, only a minority provided evidence of PCV-3 presence within lesions, suggesting the potential attribution of this virus to the etiology of the observed clinical condition [2,18,21]. The clinical significance of PCV-3 is still under investigation.

The virus has also been found in asymptomatic pigs, indicating its potential to circulate within pig populations without causing overt clinical signs [22]. Additionally, this further suggests that the development of PCV-3-related diseases and their pathogenesis may involve yet unknown factors.

Myocarditis, nephritis, and periarteritis were prevalent lesions documented in numerous reports, encompassing both field observations and experimental infection studies. Arteritis and periarteritis observed in pigs infected with PCV-3 varied from localized to systemic [1,2,21,23,24,25]. Notably, experimental infections revealed the presence of arteritis in cardiac, renal, mesenteric, and intestinal tissues [23,26,27].

While a formal case definition has been established for systemic disease (PCV-3-SD) and reproductive disorders (PCV-3-RD), the actual prevalence of these clinical-pathological conditions and their economic significance remains uncertain [28]. Even with well-defined diagnostic criteria for the two diseases linked to PCV-3, reaching a conclusive diagnosis remains challenging due to several factors. Detecting PCV-3 within lesions is mainly limited to in situ hybridization (ISH) due to the absence of commercially available reliable antibodies for immunohistological analysis of formalin-fixed, paraffin-embedded (FFPE) tissues. However, the cost of ISH poses a constraint for routine use. Additionally, clinical outcomes related to PCV-3 are nonspecific, requiring consideration of various pathogens. Histopathological expertise is crucial as lesions in PCV-3-SD and PCV-3-RD can be subtle, necessitating a level of familiarity to suspect these conditions. Conversely, there are extensive reports of viral circulation in swine populations without apparent disease, indicating that subclinical infection might be the prevailing outcome. However, the extent of its impact on productive parameters remains unclear [5,9] and the knowledge of clinicopathological and epidemiological aspects of PCV-3 infection and the pathogenic effect of the virus is limited.

The aim of the present study was to investigate the occurrence of PCV-3 in pigs from Switzerland using real-time PCR (qPCR) in frozen and FFPE samples and to gain data about the epidemiology of the virus in the country. Furthermore, we aimed to detect PCV-3 by ISH as part of the project’s objective. The samples were obtained from retrospective diagnostic cases involving piglets exhibiting histological lesions compatible with PCV-3-SD.

## 2. Materials and Methods

### 2.1. Retrospective Histopathological Evaluation of Lesions Consistent with PCV-3-Associated Diseases

This study was performed on selected cases of suckling and weaning piglets (up to 12 weeks of age) that underwent a diagnostic post-mortem examination at the Institute of Veterinary Pathology (IVPZ), Vetsuisse Faculty, University of Zurich, between 2020 and 2023. These investigations were part of the PathoPig program, which comprises state-subsidized necropsies at pathological laboratories initiated by the Swiss Federal Food Safety and Veterinary Office (FSVO) in order to improve disease diagnosis and therefore early detection of (re-)emerging animal diseases and zoonoses. Tissue samples, frozen samples, and formalin-fixed tissues were collected as part of a routine diagnostic investigation. Post-mortem examination was performed due to various clinical signs, with diarrhea, wasting, and sudden death being the most frequently reported signs by field veterinarians.

Hematoxylin and eosin (H&E)-stained tissues were re-evaluated histopathologically to identify microscopic lesions indicative of PCV-3-SD, such as vasculitis, periarteritis, and arteritis in the blood vessels of the mesenteric lymph nodes and associated vascular plexus, kidney, and heart.

### 2.2. Real-Time PCR for PCV-3

PCR establishment. A PCV-3-specific PCR targeting a 113 base pair (bp) fragment of the conserved rep gene was established according to the described method [29]. Table 1 lists the primers and probes used in this study, and Table 2 includes the master mix prepared per sample.

PCR amplification was performed on the 7500 Fast Real-Time PCR System (Applied Biosystems, Waltham, MA, USA) using the following cycling protocol consisting of 95 °C for 7 min and 45 cycles of 95 °C for 10 s and 60 °C for 30 s as described [29]. Each run included water as a negative and a standard curve as a positive control. The standard curve ranged from 10^0^ to 10^8^ copies per reaction and consisted of recombinant plasmid that contained the target sequence. Specifically, the target sequence was amplified from the DNA of a PCV-3-positive lymph node, purified with GeneJET PCR purification kit (Invitrogen, ThermoFisher Scientific, Carlsbad, CA, USA) and cloned into TOPO TA vectors with the TOPO TA Cloning Kit (Invitrogen, ThermoFisher Scientific, Carlsbad, CA, USA) according to manufacturer’s recommendations. The plasmid was amplified and purified using first the Qiagen Plasmid Mini Kit (Qiagen, Hilden, Germany) followed by linearization using HindIII-HF (New England BioLabs, Ipswich, MA, USA) and the QIAquick PCR Purification kit Qiagen, Hilden, Germany), each according to manufacturer’s instructions.

Testing of samples. Tissue samples (25–35 mg) were subjected to DNA extraction with the Qiagen^®^ DNeasy Blood and Tissue kit (Qiagen, Hilden, Germany) according to manufacturer’s instructions. Nucleic acid was extracted from FFPE tissues with the QIAamp DNA FFPE Tissue kit (Qiagen, Hilden, Germany) as instructed by the manufacturer. The eluted DNA was quantified using a NanoDrop^®^ 2000 (Thermo Fischer Scientific Inc., Bartlesville, OK, USA) following the manufacturer’s instructions. The DNA was either used directly or stored at −20 °C until further use. Each sample was tested in duplicate by qPCR.

### 2.3. Tissue Microarray (TMA)

Glass slides corresponding to the cases described above were retrieved from the tissue archives at IVPZ and reviewed to select appropriate candidate blocks.

Briefly, representative vasculitis areas were selected microscopically on HE-stained sections of heart, kidney, and lymph node and encircled with a permanent marker. An electronic spreadsheet was created to document the identification numbers of the tissues along with their locations and coordinates on the arraying device. The paraffin block was aligned with the slide to identify the marked areas. A hollow needle was then used to extract cylindrical tissue cores, each measuring 2 mm in diameter, from the designated areas of interest in the paraffin-embedded tissues. These tissue cores were inserted into a pre-perforated recipient paraffin block arranged in a 2 mm spaced array pattern, with 4 cores across the *x*-axis and 6 cores down the *y*-axis. Finally, sections of 4 μm thickness were cut from the TMA blocks using a microtome and mounted on microscope slides for ISH. Additionally, 2 μm sections were stained with HE for standard histological analysis.

### 2.4. In Situ Hybridization

From one selected case, consecutive sections (4 µm) of lymph node were prepared and tested with an RNAscope^®^ oligoprobe for Ubiquitin C (*Sus scrofa*-UBC) to confirm RNA preservation and quality, following the manufacturer’s (Advanced Cell Diagnostics, Newark, CA, USA) protocol. These yielded good UBC signals; hence, consecutive sections were then subjected to RNA-ISH for PCV-3 (Circoviridae SFpork/USA/2010 isolate; SFpork8 Rep protein-like gene complete sequence), using RNAscope^®^ oligoprobes (catalog no. 491021; Advanced Cell Diagnostics, Newark, CA, USA). An automated RNAscope 2.5 Detection Reagent Kit (Brown) was used according to the manufacturer’s (Advanced Cell Diagnostics, Newark, CA, USA) instructions, as well as a previously published protocol [30], with slight adjustments. Briefly, sections were heated to 60 °C for 1 h and subsequently deparaffinized. Permeabilization was achieved by incubating the section in pretreatment solution 1 (RNAscope^®^ Hydrogen Peroxide) for 10 min at room temperature (RT), followed by boiling in RNAscope^®^ 1X Target Retrieval Reagents solution at 100 °C for 20 min and washing in distilled water and ethanol. After digestion with RNAscope^®^ Protease Plus for 30 min at 40 °C, sections were hybridized with the oligoprobes at 40 °C in a humidity control tray for 2 h (HybEZ ^TM^ II Oven; ACD Advanced Cell Diagnostics). Thereafter a serial amplification with different amplifying solutions (AMP1, AMP2, AMP3, AMP4: alternating 15 min and 30 min at 40 °C) was performed. Between each incubation step, slides were washed with a washing buffer. They were subsequently incubated with AMP 5, AMP 6, and DAB at RT for 30 and 10 min, respectively. Hematoxylin served to counterstain the sections, which were then dehydrated with graded alcohol and xylene and coverslipped. The specificity of labeling was further confirmed by using a negative control probe (DapB, catalog no. 310043; Advanced Cell Diagnostics, Newark, CA, USA).

## 3. Results

### 3.1. Histopathological Results

**Figure 1:**
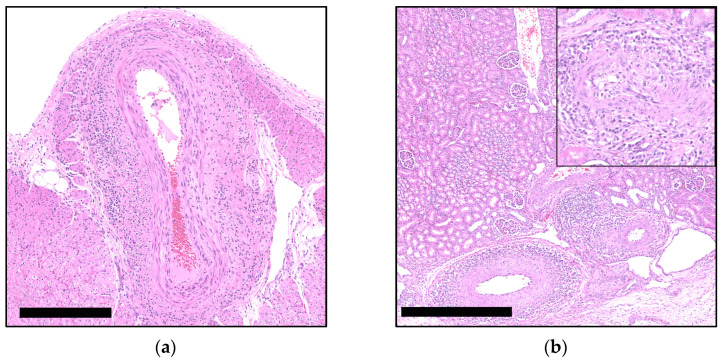
Examples of histopathological cardiac and renal lesions associated with PCV-3-SD: (**a**) Severe lymphoplasmacytic (peri-)arteritis in an epicardial artery, H&E. Bar 250 µm; (**b**) Severe lymphoplasmacytic (peri-)arteritis in multiple arteries in the renal pelvis, H&E. Bar 500 µm. Insert: Mononuclear infiltration of the arterial wall.

**Figure 2:**
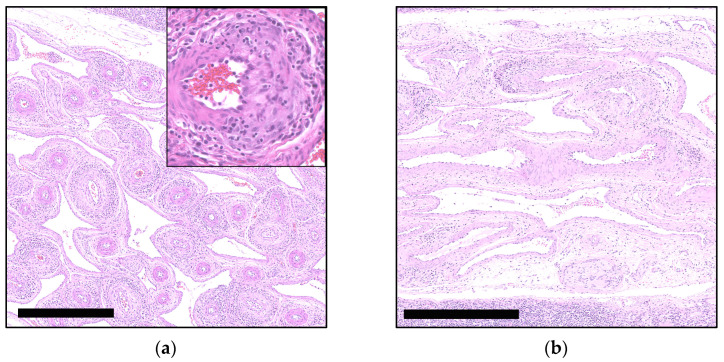
Examples of histopathological lesions of the mesenteric vascular plexus associated with PCV-3-SD and periarteritis score: (**a**) Severe (++; ≥20% affected arteries) lymphoplasmacytic and histiocytic (peri-)arteritis in mesenteric arteries, H&E. Bar 500 µm. Insert: Mononuclear infiltration in tunica adventitia; (**b**) Mild (+; ≤20% affected arteries) lymphoplasmacytic and histiocytic (peri-)arteritis in mesenteric arteries, H&E. Bar 500 µm.

Of a total of 323 re-evaluated archival cases, 19 (6%) exhibited PCV-3-SD compatible lesions in one or more of the designated organs. These cases predominantly manifested perivascular and, less frequently, vascular lymphoplasmacytic to lymphohistiocytic infiltrates within the media and adventitia layers of small and medium-caliber arteries (Figure 1). The affected vessels were primarily observed in mesenteric arteries adjacent to the mesenteric lymph node (15 out of 19, 79%), followed by the heart (9 out of 19, 47%), and the kidney (6 out of 19, 32%). Four of the cases demonstrated equally pronounced vascular lesions in all three organs, while seven cases exhibited lesions in both the lymph node and heart, five exhibited lesions in both the heart and kidney, and four exhibited lesions in both the lymph node and kidney. Notably, kidney and heart did not exhibit vascular lesions independently, whereas eight cases solely exhibited lesions in the lymph node. The lesions were categorized based on the intensity of the inflammatory infiltrates and the extent of the lesions, with the following grading scale: absence (−) of inflammatory infiltrates, mild (+) with less than 20% of vessels affected, and severe (++) with more than 20% affected (Figure 2). Apart from vascular lesions, most of the organs exhibited no additional PCV-3-related lesions, such as myocarditis, as described in the literature [30,31]. However, some cases did show lesions corresponding to the clinical problems observed at submission.

### 3.2. qPCR Results

All 19 cases with PCV-3-SD lesions underwent qPCR analysis using available frozen tissues or FFPE samples. Frozen mesenteric lymph node was available in 17 out of 19 cases. Frozen kidney tissue was available in 11 cases and heart tissue in 4 cases. When frozen material was not available, qPCR was performed on FFPE slides containing the organs of interest. Overall, nine cases (47%) tested positive in one or more organs (Table 3). Among the positive cases, the lymph nodes consistently exhibited the highest viral load, ranging from 15 copies/100 ng DNA to approximately 28,000 copies/100 ng DNA (Table 4).

### 3.3. In Situ Hybridization Results

**Figure 3:**
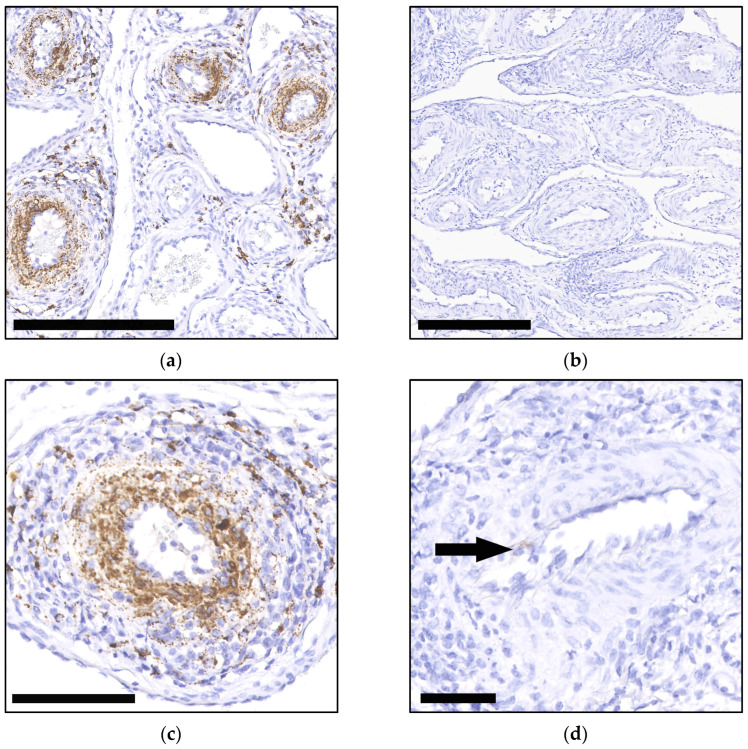
Comparative results of ISH on the mesenteric vascular plexus between a positive control case (**a**,**c**) and the selected archival case (**b**,**d**), hematoxylin counterstain: (**a**) Marked PCV-3-ISH-RNA-positive signal in multiple arteries. Bar 250 µm; (**b**) PCV-3-ISH-RNA no to weak signal. Bar 250 µm; (**c**) Marked PCV-3-ISH-RNA-positive signal (brown) in the arterial wall. Bar 50 µm; (**d**) PCV-3-ISH-RNA-weak-positive signal (arrow) in the vascular wall of an artery. Bar 100 µm.

ISH was performed on a selected archival case that had tested positive for PCV-3 by qPCR and exhibited the highest viral load among all the positive cases. The ISH results were then compared to a positive control case known to exhibit the PCV-3 ISH signal. Contrary to the qPCR results, the ISH analysis yielded negative results in the tissue of the selected archival case. No specific hybridization signal was detected in the arteries of the mesenteric vascular plexus, the heart, or the kidney. The positive control case, however, exhibited strong hybridization signals in the corresponding tissues, confirming the effectiveness and specificity of the ISH probe and technique (Figure 3).

## 4. Discussion

This study provides a retrospective analysis of PCV-3 in Switzerland using 19 cases that exhibited PCV-3-SD compatible lesions in one or more of the designated organs, presenting the first documentation of this virus within the Swiss swine population. By examining archival cases of suckling and weaner piglets, this investigation provides valuable insights into the prevalence in Switzerland and histopathological characteristics of PCV-3-SD.

In this study, we could show that a PCV-3-specific qPCR targeting the *rep* gene established for fresh tissue material [29] can be reliably applied for FFPE material, which shows similar copy numbers to frozen material. This methodological advancement enhances diagnostic accuracy and reliability in identifying PCV-3 infections, particularly in the context of post-mortem diagnostics. The application of ISH on selected qPCR-positive cases aimed to validate molecular findings but encountered significant challenges. Our study reported negative ISH results, which were attributed to prolonged tissue fixation in formalin for up to several years. This extended fixation period can compromise nucleic acid integrity and hinder hybridization efficiency.

Histopathological reassessment focusing on lymphohistiocytic and plasmacellular (peri-)arteritis, known to be the main lesions in PCV-3-SD, identified PCV-3-SD lesions in 6% of cases, with 47% testing positive for PCV-3 in one or more organs by qPCR. In another retrospective study [30], all animals displaying vascular lesions histologically were also tested positive by qPCR. In contrast, our study found that less than 50% of the cases showing lesions suggestive of a PCV-3-SD tested positive by qPCR. This discrepancy could be attributed to the likelihood of sampling frozen material during necropsy without PCV-3-associated lesions or the contribution of other pathogens to lesion development.

In our study, apart from the described vascular lesions, most of the organs exhibited no additional PCV-3-related lesions, such as myocarditis, PDNS, and lymphoid depletion, as previously described [1,30,31]. The animals did also not display clinical symptoms related to PCV-3-SD. Furthermore, the histopathological examination highlighted the variability in lesion severity and distribution among cases and tissues. For instance, while some cases exhibited pronounced vascular involvement across multiple organs, others showed lesions restricted to specific anatomical sites. Furthermore, there was no correlation between the severity and the distribution of the histological lesions among the organs and the amount of viral DNA detected by qPCR. This variability underscores the heterogeneous nature of PCV-3 infection, which may be influenced by factors such as the host immune response and concurrent infections. Notably, in our study, there was no association between the specific cause of submission of the cases and the presence of PCV-3 infection. Additionally, there were no discernible age-related patterns since PCV-3 infection was observed across all tested age groups. These findings emphasize the complexity of PCV-3 pathogenesis and suggest that the virus may interact with a variety of host and environmental factors. The absence of a clear correlation between clinical presentation, age, and PCV-3 infection highlights the multifactorial nature of the disease and the potential for a wide range of influences on its manifestation and progression. This complexity necessitates further research to elucidate the mechanisms underlying PCV-3 infection and its variable clinical outcomes across different age groups and conditions.

Like in other retrospective studies [1,13,21,30,32], the qPCR analysis of archival tissue in our study confirmed the presence of PCV-3 in various tissues, highlighting its systemic dissemination and varying viral loads across affected organs. These findings underscore the utility of molecular diagnostics in elucidating the distribution and intensity of PCV-3 infection within affected pig populations.

Furthermore, PCV-3 appears to have circulated in Switzerland at least since 2020, as evidenced by the oldest positive cases identified in our study. However, further investigations are warranted to determine whether the virus is also present in older cases. To enhance future research, avoid the limitations encountered in this study, and provide more accurate results, we recommend employing multiple probes simultaneously for ISH to improve detection sensitivity in archival samples. Additionally, as performed by other authors [18], using probes derived from DNA fragments amplified by qPCR increases the reliability of RNA detection. The literature indicates that PCV-3 has an ancient origin [33], suggesting a potential presence in earlier cases that has not yet been systematically studied. Continued research will be essential to comprehensively understand the historical and ongoing prevalence of PCV-3 in the Swiss swine populations.

## 5. Conclusions

In conclusion, this comprehensive retrospective analysis has provided documentation of PCV-3 within the Swiss swine population through an in-depth examination of archival cases encompassing suckling and weaner piglets. This study represents the first description of PCV-3 in Switzerland, highlighting its emergence and dissemination within the country’s swine herds. The development and successful implementation of a qPCR protocol for detecting PCV-3 in both frozen tissue and FFPE samples represent significant methodological advancements, enhancing national diagnostics across diverse sample types, particularly for post-mortem diagnostics. Despite challenges encountered with ISH validation due to prolonged formalin fixation compromising nucleic acid integrity, our study underscores the utility and limitations of molecular techniques in confirming PCV-3 infections.

## Figures and Tables

**Table 1:** Primers and probes used for the detection of PCV-3 [29].

Primer/Probe	Oligonucleotide	Assay
PCV3_353_F	5′-TGACGGAGACGTCGGGAAAT-3′	qPCR
PCV3_465_R	5′-CGGTTTACCCAACCCCATCA-3′	qPCR
PCV3_418_probe	5′-GGGCGGGGTTTGCGTGATTT-BHQ1-3′	qPCR

**Table 2:** Master mix prepared per sample.

Reaction System	Volume in μL (total volume 20 μL)
TaqMan™ Fast Universal PCR Master Mix ^1^	10
Primer F	1.2
Primer R	1.2
Probe	0.6
DNA template	2
H_2_O	5

^1^ TaqMan™ Fast Universal PCR Master Mix (2X), no AmpErase™ UNG; Invitrogen ThermoFisher Scientific, catalogue number: 4367846.

**Table 3:** Summary of histological graded lesions (HgL) compared with the qPCR results in different tissues for the qPCR-positive cases.

Case Nr.	Age (w)	mLN ^1^ HgL/qPCR	Kidney HgL/qPCR	Heart HgL/qPCR
1	1	−/+	−/+	+/+
2	12	+/+	−/−	−/−
3	8	++/+	−/+	+/−
4	8	++/+	+/−	++/−
5	2	+/+	−/−	−/−
6	2	+/+	−/−	+/−
7	7	+/+	+/−	+/−
8	0.5	+/+	+/+	+/−
9	2	+/+	−/−	−/−

^1^ mesenteric lymph node; w = weeks. Extent of the histological lesions: absence (−) of inflammatory infiltrates, mild (+) with less than 20% of vessels affected, and severe (++) with more than 20% affected. qPCR: positive (+), negative (−).

**Table 4:** Number (and percentage) of PCV-3 qPCR-positive tissue samples.

Tissue	Proportion of qPCR-Positive Samples (%)	PCV-3 Viral Load Ranges (PCV-3 Copies/100 ng DNA)
mLN ^1^	9/19 (47%)	28,599 (Case 4)—15 (Case 6)
Kidney	3/19 (16%)	2014 (Case 3)—10 (Case 8)
Heart	1/19 (5%)	49 (Case 1)

^1^ mesenteric lymph node.

## Data Availability

Data are contained within the article.
